# Suspected dog bite associated HIV horizontal transmission in Swaziland 

**DOI:** 10.4102/phcfm.v5i1.440

**Published:** 2013-08-27

**Authors:** Ganizani Mlawanda

**Affiliations:** 1 Department of Family Medicine, University of Stellenbosch, South Africa; 2 Royal Swaziland Sugar Company Medical Services, Simunye Hospital, Swaziland

## Abstract

**Background:**

Dog bites may lead to transmission of bacteria and viruses over and above tetanus and rabies. Theoretically human immunodeficiency virus (HIV), Hepatitis B and Hepatitis C may be transmitted after dog bites where transfer of blood from one victim to another occur in clinical practice HIV, Hepatitis B and Hepatitis C are not considered when making treatment decisions, nor adequate patient history taken to consider all potential risks after dog bites in succession.

**Objective:**

To present case of suspected HIV transmission after dog bites in close succession involving two HIV sero-discordant victims.

**Management and outcome:**

Management and outcome: HIV rapid test and/or HIV Ribonucleic acid (RNA) polymerase chain reaction (PCR) results for the victim(s) at presentation and a month later.

**Results:**

Two night patrol guards presented to casualty after dog bites in close succession by the same dog. They were managed according to the dog bite protocol. Thinking out of the box, the first victim was found to be HIV positive by rapid test whilst the second victim was negative based on both HIV rapid test and HIV RNA PCR. One month after the dog bites, a case of HIV sero-conversion was confirmed in the second victim despite post-exposure prophylaxis (PEP).

**Discussion:**

Although an isolated case, shouldn’t clinicians re-think the significance of HIV transmission after animal bites where there is repeated blood exposure in several people in succession?

**Conclusion:**

Clinicians should be aware of the potential of HIV, Hepatitis B and C transmission, when faced with dog bites in succession.

## Abstrait

Transmission horizontale du VIH associée à une morsure de chien suspectée au Swaziland

**Contexte:**

Les morsures de chien entraînent une transmission de bactéries et de virus, autres que le tétanos et la rage. Théoriquement, le virus de l’immunodéficience humaine (VIH), l’hépatite B et l’hépatite C peuvent être transmis après des morsures de chien lorsqu’il se produit un transfert de sang d’une victime à une autre. Dans la pratique clinique, le VIH, l’hépatite B et l’hépatite C ne sont pas pris en considération lors de la prise de décisions relatives au traitement, et aucun antécédent du patient n’est pris en compte pour envisager tous les risques potentiels après des morsures de chiens consécutives.

**Objectif:**

Présenter un cas de transmission du VIH suspectée après des morsures de chiens se succédant rapidement et impliquant deux victimes séro-discordantes.

**Gestion et les résultats:**

Les résultats du test rapide de dépistage du VIH et/ou du test d’ACP de l’ARN du VIH pour la (les) victime(s) lorsqu’elles se présentent et un mois après.

**Résultats:**

Deux patrouilleurs de nuit se sont présentés aux urgences après avoir été mordus par le même chien de manière très rapprochée. Ils ont été pris en charge conformément au protocole associé aux morsures de chiens. En sortant des sentiers battus, il a été constaté, après un test rapide, que la première victime était séropositive tandis que la deuxième victime était séronégative d’après un test rapide du VIH et un test d’ACP de l’ARN du VIH. Un mois après les morsures de chien, un cas de séroconversion a été confirmé chez la deuxième victime malgré une prophylaxie post-exposition (PEP).

**Discussion:**

Bien qu’il s’agisse d’un cas isolé, les cliniciens ne devraient-ils pas reconsidérer l’importance de la transmission du VIH après des morsures d’animaux lorsqu’une exposition au sang répétée chez plusieurs personnes se produit successivement?

**Conclusion:**

Les cliniciens devraient être conscients de la possibilité de transmission du VIH, de l’hépatite B et de l’hépatite C, lorsque l’on fait face à des morsures de chiens se succédant.

## Introduction

Dog bites accounts for 60% – 90% of all animal bites, whilst cat bites and human bites account for 5% – 18% and 4% – 23% respectively.^1^ Dogs seldom bite multiple victims and young adult males are the most common casualty.^1,2^

The HIV pandemic in sub-Saharan Africa affects nearly one in four young adults.^3^ Thus, the at-risk population for both HIV infection and dog bites is the same. Furthermore, there is a theoretical potential of HIV, hepatitis B and hepatitis C (apart from the usual dog-bite-associated pathogens) being transmitted following dog bites when there is a transfer of blood from one victim to another.^1,2,3^

To date, there are no studies or case reports related to HIV transmission and dog bites. This case study, the first of its nature, described a *possible association* of HIV transmission following dog bites involving multiple victims. Direct causality was problematic to infer. One obvious reason was that the newly-infected victim might have acquired HIV from other sources, an example being from his sexual partner(s).

 Whilst not authoritatively conclusive, it acted as an eye-opener to the potential of such a possibility, thus stimulating further research in this area and increasing clinician awareness of other sources of horizontal HIV transmission.

## Ethical considerations

Institutional approval was obtained prior to authorship of this case study. In addition, no mention of patients’ names is made in order to protect identities. 

## Case presentation

 Two night patrol security guards (Mr X and Mr Y) presented to the casualty department at Royal Swaziland Sugar Company (RSSC) Simunye Hospital in Swaziland, two hours after having suffered dog bites in rapid succession from the same dog. The tetanus immunisation status of both men was not up to date, and there was no history of known immunosuppressive diseases in either case.

On examination they were both bleeding from the bite sites but were haemodynamically stable. They both suffered severe bite wounds on the lower limbs, and full-thickness muscle damage without tendon or nerve involvement. Apart from the findings mention above, the rest of the examination was unremarkable.

The local dog bite protocol was followed. Bleeding was controlled and wound toilet performed. The surgical team was involved in wound care, and there was a need for debridement at a later stage. Tetanus and rabies vaccinations plus appropriate antibiotic cover were given.

One of the casualty primary care nurses, thinking outside the ‘protocol’, wondered if it was vital to do HIV testing, since the victims were bitten in succession. The suggestion made scientific logic. After pretest counselling HIV testing was done using two rapid tests (fourth-generation Determine and Unigold). The first victim (Mr X) turned out to be HIV-positive on both tests, whilst the second victim (Mr Y) tested HIV negative. The HIV-negative status for Mr Y was further confirmed by HIV Deoxyribonucleic acid (DNA) polymerase chain reaction (PCR). In accordance with latest guidelines, HIV post-exposure prophylaxis (PEP) was initiated within four hours, using a three-drug regimen tenofovir (TDF) 300 mg once daily, emtricitabine (FTC) 150 mg once daily, and efavirenz (EFV) 600 mg at night for four weeks), which was well tolerated. In addition, PEP-related precautions and adherence were reinforced. The adherence rate was 82% (23/28) based on a pill count done at the end of the 28 days. Hepatitis B and hepatitis C screening were negative in both cases. The HIV-positive guard, Mr X, was subsequently referred for further management at the local HIV clinic.

After completion of HIV PEP, HIV rapid tests were repeated in Mr Y, and were positive. An HIV RNA PCR was performed and confirmed a very high viral load of 7 log RNA copies/mL, a hallmark of acute HIV infection.

Subsequent post-test counselling and occupational compensation-related issues (in keeping with local policies) were discussed with the affected guard, Mr Y, as well as enrolment into chronic HIV care. The bite wounds healed uneventfully in both Mr X and Mr Y. 

## Discussion

Bites are common, with dog bites accounting for 60% – 90% of bites, cat bites for 5% – 18% and human bites for 4% – 23%.^1,2^ Most dog bites occur in young adult males. Bites often result in broken skin and/or involvement of subcutaneous structures. HIV prevalence in most Southern African countries hovers above 25%.^3^ Dogs may bite multiple victims in the same incident or in close succession, hence the potential of blood transfer from one victim to another. One report found that dogs are more vicious during full moon and often bite multiple casualties during this period.^4^ With dog bites there is also potential bacterial (staphylococcus; streptococcus; eikenella; pasteurella; proteus; klebsiella; haemophillus; enterobacter; *Capnocytophaga canimorsus*; bacteroides)^5^ and viral (HIV, hepatitis B, hepatitis C)^1,2,5,6^ transmission, especially with repeated blood exposure in several people at once.

Unfortunately only a small proportion of bite victims seek medical attention, according to a United Kingdom study.^1^ There are no statistics available for the Southern African setting in relation to medical attention-seeking behaviour following animal bites. Several articles have highlighted the possibility of non-sexual horizontal HIV transmission in Africa as a contributor to the high HIV prevalence.^6,7,8,9^ Whilst unlikely, bite-related HIV transmission (especially in cases where there is repeated blood exposure amongst several people at once) may be a potential contributor to non-sexual HIV transmission in high HIV prevalence regions, especially Southern Africa, considering that most bites go unreported.^1,2,3,6,7,8,9^

This case was an example of suspected HIV transmission following dog bites in two victims at the same incident. The risk of animal bites-related HIV transmission (especially in cases where there is repeated blood exposure amongst several people at once) is undocumented in the literature. In clinical practice, the possibility of HIV exposure through dog bites is rarely considered, attention being mainly on prevention of rabies, tetanus and wound infection. This case highlights the importance of adequate history taking and increasing clinicians’ awareness of the possibility of HIV transmission in cases of multiple victims suffering bites from the same dog in close succession, especially in high HIV prevalence areas. The cost of preventing one case of HIV transmission following a bite greatly outweighs the eventual cost of a lifetime of highly active antiretroviral treatment (HAART) and HIV-related morbidity.

In general, the management of dog bites involve taking of a detailed history; assessment of the bite extent, including muscle, tendon and nerve damage; and assessment of the need for tetanus and rabies vaccinations. For rabies prevention the most commonly used schedule recommends rabies vaccination for bites involving broken skin (grade 2) and those with skin penetration (grade 3).^10^ Thus, if there is rabies exposure risk and bites involve multiple victims, clinicians should always consider HIV transmission risk and the need for HIV PEP.

The exact reason for the failure of HIV PEP and subsequent sero-conversion in the case of Mr Y could be explained in many ways. The choice of PEP was based on the latest recommendations for a three-drug combination as opposed to two drugs.^11^ In addition, the use of TDF and/or FTC and/or EFV is known to be efficacious and well tolerated and has less side-effects.^11^ This patient did not report any undesired effects, despite literature suggesting that antiretroviral-related side-effects are more common and severe in HIV-negative-exposed individuals than in HIV-positive ones initiated on treatment.^11,12^ However, his adherence of 82% was sub-optimum and might have been the cause of PEP failure. In addition, the inherent failure risk of HIV PEP failure or the possibility of yet unknown interactions between the cocktail of bite-related therapeutic interventions (debridement, anti-rabies, anti-tetanus and antibiotics) and the HIV PEP regimen (TDF and/or FTC and/or EFV) might have led to the failure. Unfortunately there are no documented or scientifically plausible pharmacological interactions between the PEP regimen used and the antibiotic, anti-rabies or anti-tetanus vaccinations used.

Besides PEP failure, potential reasons for sero-conversion include the extent of the bite injuries; direct and indirect blood transfer between involved patients; the possibility of being in the ‘window period’ at the time of the bite; and transmission from ‘non-bite-related sexual exposure’. The patient denied any possible sexual contact during the time he was on PEP and that can, in theory, aid in excluding sexual transmission. The possibility of direct (for example, incidental blood contact at the scene) or indirect blood transfer (for example, shared surgical instruments) was ruled out by a thorough audit of events surrounding the incident and the strict infection control measures which are the norm at the institution. This case acts as a stimulus for further reporting of cases of dog bite-related HIV transmission, and to increase clinicians’ awareness of the possibility of such. In addition, it was an eye-opener as to PEP failure as a result of the real-life sub-optimal adherence to PEP, and a stimulus for further observational studies of PEP adherence.

One weakness of this case study was the lack of definite scientific proof by molecular and/or biological techniques to ascertain similarity between the Mr X and Mr Y’s HIV genotypes: this could not be done when the sero-conversion occurred, as Mr X was lost to follow-up. However, using a balance of probability approach, there more than likely was an association between the dog bite and the subsequent HIV transmission.

In conclusion, exposure to HIV occurs in a bewildering variety of situations, and animal bites with repeated blood exposure in several people at once is no exception. So clinicians – be ever alert of HIV risk, and use clinical common sense on a case-by-case basis. 
